# Isolation, complete genome sequencing and in silico genome mining of *Burkholderia* for secondary metabolites

**DOI:** 10.1186/s12866-022-02692-x

**Published:** 2022-12-30

**Authors:** Khorshed Alam, Yiming Zhao, Xuefei Lu, Kai Gong, Lin Zhong, Jinfang Hao, Md. Mahmudul Islam, Saiful Islam, Geng Li, Youming Zhang, Ruijuan Li, Aiying Li

**Affiliations:** 1 Helmholtz International Lab for Anti-Infectives, State Key Laboratory of Microbial Technology, Shandong University-Helmholtz Institute of Biotechnology, Shandong University, Qingdao, 266237 People’s Republic of China; 2 Department of Microbiology, Rajshahi Institute of Biosciences (RIB), Affi. University of Rajshahi, Rajshahi, 6212 Bangladesh; 3 Bangladesh Council of Scientific and Industrial Research (BCSIR), Chattogram Laboratories, Chattogram, 4220 Bangladesh

**Keywords:** Burkholderia, Natural products, Genome mining, Biosynthetic gene cluster

## Abstract

Recent years, *Burkholderia* species have emerged as a new source of natural products (NPs) with increasing attractions. Genome mining suggests the *Burkholderia* genomes include many natural product biosynthetic gene clusters (BGCs) which are new targets for drug discovery. In order to collect more *Burkholderia*, here, a strain S-53 was isolated from the soil samples on a mountain area in Changde, P.R. China and verified by comparative genetic analysis to belong to *Burkholderia*. The complete genome of *Burkholderia* strain S-53 is 8.2 Mbps in size with an average G + C content of 66.35%. Its taxonomy was both characterized by 16S rRNA- and whole genome-based phylogenetic trees. Bioinformatic prediction in silico revealed it has a total of 15 NP BGCs, some of which may encode unknown products. It is expectable that availability of these BGCs will speed up the identification of new secondary metabolites from *Burkholderia* and help us understand how sophisticated BGC regulation works.

## Introduction

The prevalence of drug-resistant pathogens has been a serious problem and effected the human life and agriculture. The World Health Organization (WHO) estimates ten million deaths by 2050 if multi-drug resistant (MDR) infections are not appropriately managed [1, 2]. All major antibiotic classes have been found to have antimicrobial  resistance, and the number of candidates for novel antibiotics is dwindling. Hence screening novel antibacterial compounds is critical for new drug discovery [3].

Microbial natural products are the important sources of drug discovery because of their structural diversity to make up more than 75% of antibiotics [4, 5]. The 99.99% rediscovery rate in traditional discovery pipelines of natural products is a big drawback [6]. However, the last decade has been a revival time for natural product discovery which was fueled by advances in analytical chemistry, bioinformatics, and whole genome sequencing [7].

Microbial genome sequencing revealed that they contain huge sources of cryptic BGCs, which have a larger capability to produce secondary metabolites. Availability of whole genome sequences and synthetic biology-inspired tools/approaches make it possible to utilize these BGCs to develop new chemicals with new structures, new activity and new targets [8].

Modern natural product discovery relies on, to a higher extent, on the microbial genome sequencing and computer mining for BGCs. Next stages include selecting unique BGCs, cloning and expressing selected BGCs in an optimal heterologous host or activating in situ silent BGCs. This pipeline (genome mining of NPs) takes less time on dereplication and streamlines NP discovery via the use of advanced computational, microbiological and synthetic biological approaches, to more extents, compared to traditional screening methods.

Most members of *Burkholderia* are well-known as pathogens to their hosts (plants or human) and now 44 members in this genus have been identified [9]. In recent years, many species of *Burkholderia* have been found to have the ability to excrete a range of secondary metabolites, including antibacterial, anticancer, herbicidal, and insecticidal chemicals that can act as bioremediation, biocontrol and plant growth promotion agents [10, 11].

More recently, the increasing data of *Burkholderia* genome sequences have shown a vast reservoir of NPs, such as non-ribosomal peptides (NRPs) and polyketides (PKs), with various pharmacological functions [12]. Many silent BGCs in *Burkholderia* genomes remain unexplored as potential drug development targets. Using genome mining approaches, many compounds, such as bolagladins/glidochelins, gladiofungins, thailandepsins/burkholdacs, romidepsin (FK228) and so on, were discovered from *Burkholderia* [13].

Due to the restrictive growth conditions, only a limited number of *Burkholderia* species have been isolated and identified as having NP BGCs or NP producers. Thus, the isolation of more species in *Burkholderia* from various environments and high-quality sequencing of *Burkholderia* genomes still are necessary for multi-omics research, which aids in the understanding of BGC regulation and rationally designing biosynthetic pathways of NPs [14, 15].

The purpose of this research was to determine the potential of a *Burkholderia* strain S-53 obtained from a small mountain area, which showed a quicker growth rate among three species of *Burkholderia*. Its genome was sequenced and analyzed for the presence of putative NP BGCs. Our data revealed this strain contains a substantial number of BGCs, indicating that its potential capability of producing new chemicals with biological activity.

## Materials and methods

### Isolation and characterization of *Burkholderia*

We collected soil samples from a small mountain (location: Tiesi Gang in Zoushi Town, 29.12755 N,111.564903E) in Changde City, Hunan Province, P.R. China using sterilized spoons. Soil samples were pretreated by drying at room temperature and then soaked in PBS buffer (10 mL PBS/g soil). Pretreated samples were serially diluted in PBS and seeded onto solid CYMG (8 g/l Casein peptone, 4 g/L Yeast extracts, 4.06 g/L MgCl_2_·2H_2_O, and 10 ml/L 50% Glycerin) medium, then cultivated  at 28 ℃ for 2 days. Whitish colonies were analyzed by colony PCR for 16S rRNA amplification with universal primers 27F(5’-AGAGTTTGATCCTGGCTCAG-3) and 1492R (5’-TACGACTTAACCCCAATCGC) under the standard PCR conditions (95℃ for 5 min, then 30 cycles of 94℃ for 1 min, 55–58℃ for 1 min and 72℃ for 90 s), followed by sequencing in Sangon Biotech (Shanghai) to pick out *Burkholderia* species. Morphological features of S-53 were recorded when cultivating on CYMG agar plates and molecular taxonomic approaches via TrueBac™ ID system and Type Strain Genome Server (TYGS)) were used to characterize the resultant isolates.

### Measurement of the growth curve of S-53

S-53 was inoculated into CYMG microwell plates (400 μ L CYMG broth in each well) using 15 wells as parallel groups, and cultivated at 30℃ for 30 h. During cultivation, OD_600_ values for each well were recorded once at 1 h interval. Taken the OD_600_ value of each parallel well at the 0 h as the blank control, the difference (OD_600/n–h_ -OD_600/0-h_) between the OD_600_ at each time-point (n- h) and OD_600_ at 0 h was calculated to represent the growth of S-53. Using the average values of OD_600/n–h_ -OD_600/0-h_ as Y-axis and time-point per h as X-axis, the growth curve of S-53 was obtained.

### Extraction of high molecular weight genomic DNA

*Burkholderia* strain S-53 was inoculated into 50 mL of CYMG liquid culture medium with glass beads (3 ± 0.3 mm diameter) in a 250 mL baffled flask and cultured for 24 h at 30 °C in a 200-rpm orbital shaker. To extract genomic DNA (gDNA), 50 mL cultivated cells were collected during the exponential growth phase and washed twice with the same amount of 10 mM EDTA followed by 45 min at 37 °C with lysozyme (10 mg /mL). gDNA for gram negative bacteria was extracted using TIANamp Bacterial DNA kit from Tiangen Biochemical Technology (Beijing) Co., Ltd, according to the instructions from the manufacturer. We determined the quality and amount of extracted gDNA samples using 1% agarose gel electrophoresis on Nanodrop (Thermo Fisher Scientific, Waltham, MA, USA).

### de novo Genome sequencing, assembly and annotation

To get fine sequence data, gDNA of S-53 was submitted to GENEWIZ Biotechnology Co., Ltd in Tianjin, China for genome sequencing with two methods:

- For Illumina sequencing, firstly, DNA was fragmented into around 500 bp, repaired for blunt ends, and then modified with the base "A" through the 3' end, so that the DNA fragments can be connected to the linker with the "T" base at the 3' end. The target fragment ligation product is recovered, and then PCR is used to amplify the DNA fragments with adapters at both ends, and finally the qualified library is used for cluster preparation and sequencing.
- For PacBio sequencing, 5–10 μg genomic DNA was sheared into 10–15 kb fragments using a g-TUBE device. Then library was constructed using the SMRTbell® Express Template Preparation Kit 2.0. The PCR products obtained using library DNA as templates were cleaned up and validated using an Agilent 2100 Bioanalyzer. Next, the qualified libraries were sequenced with pair-end PE150 on the illumina HiseqXten/Novaseq/MGI2000 System or on Sequel II sequencing platform.

The library sequenced were assembled using HGAP4/Falcon of WGS-Assembler 8.2 [16–21], then recorrected with software Pilon using previous illumine z data or Quiver.

Finding coding genes was conducted using the Prodigal [22]/Augustus [23] gene-finding software while detection of transfer RNAs (tRNAs) was done using the program tRNAscan-SE [24] with default parameter settings. rRNAs were identified by using Barrnap. Other RNAs were identified by rfam database. By BLAST using National Center for Biotechnology Information (NCBI) NR database, the coding genes were annotated (screening conditions were displayed in Table 1).

**Table 1:** Features of *Burkholderia* strain S-53

Feature	Value
Size (bp)	8,254,067
G + C percentage	66.35%
Coding region(bp)	7,142,220
Total genes	7392
RNA genes	153
Protein-coding genes	7239
Protein-coding genes with enzymes	1897
Genes assigned to COGs	5707
COG clusters	2130
Genes with signal peptides	863
Genes with transmembrane helices	1717
Number of contigs	3
Number of UBCG (paralogs)	92/92
N50	3,361,469 bp

GO [25] (Gene Ontology) database and KEGG [26] (Kyoto Encyclopedia of Genes and Genomes) database were used for analyzing functions of genes and annotating the pathways. The database of COG/KOG [14] (Clusters of Orthologous Groups) was used for phylogenetic classification of proteins.

### Phylogenetic analysis

Two methods were used for phylogenetic analysis of S-53:

(i) Whole genome-based taxonomic analysis was conducted using the Genome BLAST Distance Phylogeny approach (GBDP) by uploading genome sequence data to the Type (Strain) Genome Server (TYGS), a free bioinformatics platform accessible at https://tygs.dsmz.de [27].
(ii) A phylogenetic tree was constructed based on the 16S rRNA gene sequence of the *Burkholderia* strain S-53 and those extracted from the list of hits from EzBioCloud 16S database [28]. Evolutionary trees were established with maximum-likelihood methods [29] in MEGA X package [30]. The confidence of the tree topologies was assessed by 100 bootstrap replicates.

### Whole genome sequences for bacterial identification

Bacterial identification utilizing whole genome sequences was conducted on the TrueBac™ ID technology, a cloud-based service [31] to reveal the genuine identification of bacterial isolates using a multitude of methods.

### Comparative genomic studies/whole genome relatedness

For a whole genome-based taxonomic analysis, the genome sequence data were uploaded to the Type (Strain) Genome Server (TYGS), a free bioinformatics platform accessible at https://tygs.dsmz.de (accessed 28 December 2021). The Genome BLAST Distance Phylogeny approach (GBDP) was used to calculate dDDH (digital DNA–DNA hybridization) values and construct minimum evolution trees using TYGS [32, 27]. MEGA-X [30] was used to visualize GBDP trees. The ANI/AAI-Matrix calculator was used to calculate the average nucleotide identity (ANI) [33, 34]. The average amino acid identity (AAI) and average nucleotide identity (ANI) matrices of all conserved genes in the core genome were computed by the BLAST algorithm and visualized as heat maps for a more in-depth qualitative comparison between the genomes.

Using EZBIOCLOUD, the average nucleotide identity (ANI) of the assembled genome nucleotide files was calculated against the whole genome sequences of the strains used for 16S rRNA sequence analysis [35]. This method computes nucleotide identity through pairwise sequence alignment, yielding an overall average similarity of the genomes that is independent of sequence length.

The CGView (http://cgview.ca/) was used to generate a graphical representation of the BLAST result comparison of the available genomes to the genome of *Burkholderia* strain S-53.

### Secondary metabolite biosynthetic gene cluster prediction

As a main approach for finding and annotating genes in BGCs across the genome, antiSMASH 6 [36] combined with ClusterBlast, ActiveSiteFinder, ClusterBlast, Cluster PFam analysis, SubClusterBlast, PRISM 4 and BAGEL 4 [36] was used for discovery of BGCs in the genome of S-53 for secondary metabolites.

Particularly, BAGEL 4 was used to mine BGCs for RiPPs and bacteriocin, whereas PRISM 4 was designed for structural prediction of secondary metabolites [37]. Several database systems, including the principles of hidden Markov model (HMM) [38], BLAST algorithm [39], PFAM [40], GenBank [41], UniprotKB [42], bactibase [43], CAMPR3 [44], and the MiBig data repository [45] were used for BGC annotation. As well, NapDos was used [46] to look for KS and C domains in these genomic sequences.

## Results

### Morphological and microscopic examination and phylogenetic analysis of 16S rRNA

**Fig. 1:**
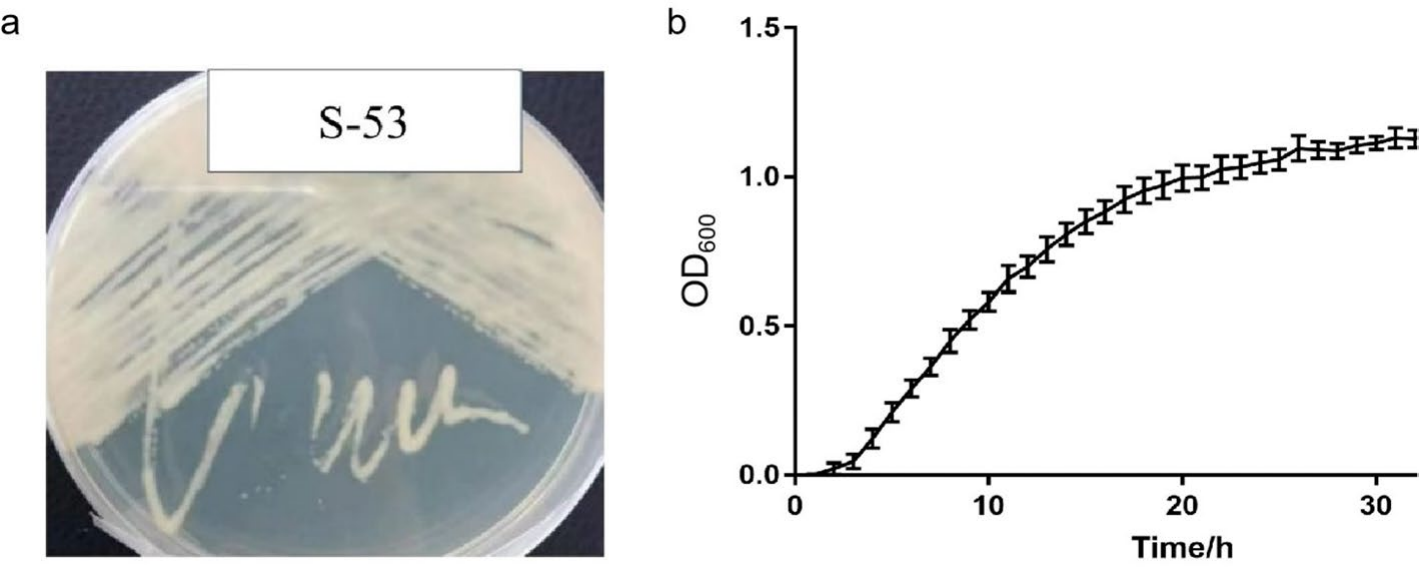
Morphology of *Burkholderia* colonies on CYMG agar medium (cultivation duration:18 h) (**a**) and the growth curve of S-53 on CYMG broth (**b**)

In order to isolate more species of *Burkholderia* from the soil samples, we incubated a serial of isolates on CYMG medium at 30℃, followed by colony PCR amplification for 16S rRNA gene. Next by 16S rRNA-based phylogenetic analysis, 3 isolates were identified as *Burkholderia,* representing different species: S-53 shared the highest gene identity of 16S rDNA (99.93%) with the type strain *Burkholderia stabilis* (NCBI Blastn). The S-53 colonies on CYMG medium were recorded (Fig. 1).

The partial 16S rDNA gene sequence of the S-53 strain, 1337 bps in length, was deposited in the GenBank nucleotide database with an accession number of OM019084.

Among three strains we isolated, we found S-53 grows more rapidly (much shorter than 18 h into its stationary stage) than other two (longer than 18 h) (Fig. 1b). Because a higher growth rate is an important feature for species of *Burkholderia* for expressing of NPs, we chose S-53 for next genome sequencing.

### Genomic features of *Burkholderia* strain S-53

The genome of *Burkholderia* strain S-53 is 8.254 Mbps in length and composed of 7239 protein-encoding genes, 63 tRNA genes, 18 rRNA genes and 72 ncRNA genes (Table 1 and Table 2).

**Table 2:** Statistics of function annotations for protein-encoding genes in S-53

Feature/database resource	Value/number
Gene_number	7239
NR	7178
KEGG	3825
GO	4473
COG	5707
CAZy	613
Pfam	6198
Swiss_Prot	5178

**Fig. 2:**
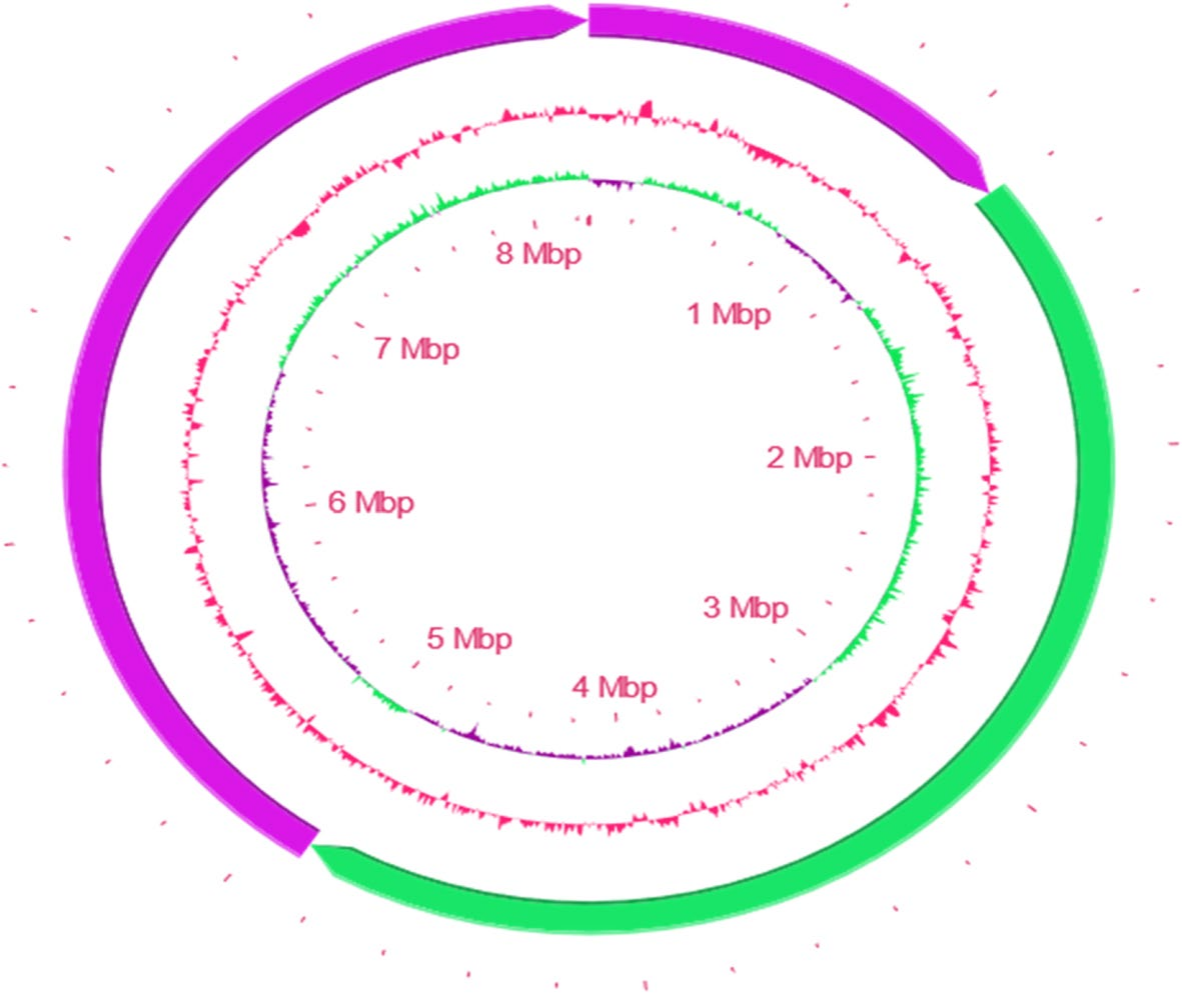
Schematic representation of the circular chromosome of *Burkholderia* strain S-53, created by CG View server (http://cgview.ca/). Circle 1 (outermost) displays the 3 Contigs while circle 2 displays the GC content plot and circle 3 (innermost) displays the GC skew. To indicate genome sizes inside and outside, the ruler was used in the chromosome map

Figure 2 showed a circular chromosome based S-53 genome sequence using CG View server (http://cgview.ca/), which is a web-based tool for comparative genomics analysis on circular genomes [47].

The genome sequence of the *Burkholderia* strain S-53 has been deposited at GenBank under the GenBank accession CP090482-CP090484.

### Bacterial strain identification by whole genome sequence and comparative genome analysis of S-53

Here, using TrueBac™ ID system [31] for bacterial identification based on whole genome sequence of S-53 strain, it could be identified as *Burkholderia pyrrocinia* (Table 3).

**Table 3:** Identification of *Burkholderia* S-53 based on whole genome sequence

Query name	Identified as	Similarity (%)	Decision	UBCG	Genome size (bp)	Taxonomy
S-53	*Burkholderia pyrrocinia*	**95.99** (G)	DEFINITIVE	92/92(0)	8,254,067	Proteobacteria;Betaproteobacteria; Burkholderiales;Burkholderiaceae Burkholderia

Further, we performed comparative genome analysis of *Burkholderia* S-53 (Table 4): the pairwise comparison of *Burkholderia* strain S-53 was recorded from TYGS [27] which is a fast-increasing discipline of genome-based taxonomy descriptions of new genera, species, and subspecies (https://tygs.dsmz.de/).

**Table 4:** Pairwise comparisons of S-53 as query strain using TYGS

Subject strain	dDDH (d0, in %)	C.I. (d0, in %)	dDDH (d4, in %)	C.I. (d4, in %)	dDDH (d6, in %)	C.I. (d6, in %)	G + C content difference (in %)
*B. pyrrocinia* DSM 10,685	**69.8**	**[65.8—73.4]**	**65.9**	**[62.9—68.7]**	**71.3**	**[67.8—74.5]**	**0.11**
*B. catarinensis* 89	58.4	[54.8—61.9]	49.6	[46.9—52.2]	57.4	[54.2—60.6]	0.05
*B. stabilis* ATCC BAA-67	53.6	[50.1—57.1]	48.8	[46.2—51.4]	53.1	[50.0—56.1]	0.07
*B. arboris* LMG 24,066	58.1	[54.4—61.6]	47	[44.4—49.6]	56.4	[53.2—59.5]	0.5
*B. seminalis* LMG 24,067	55.1	[51.5—58.5]	46.1	[43.5—48.7]	53.6	[50.5—56.7]	0.73
*B. puraquae* CAMPA 1040	57.4	[53.8—60.9]	46	[43.4—48.6]	55.6	[52.4—58.7]	0.25
*B. cepacia* ATCC 25,416	57	[53.4—60.5]	45.9	[43.4—48.5]	55.2	[52.1—58.3]	0.26
*B. reimsis* BE51	55.2	[51.7—58.7]	45.8	[43.3—48.4]	53.7	[50.6—56.8]	0.04
*B. lata* 383	54.3	[50.8—57.8]	45.7	[43.1—48.3]	52.9	[49.8—55.9]	0.08
*B. cenocepacia* J2315	53.4	[49.9—56.8]	45.6	[43.0—48.1]	52	[48.9—55.1]	0.55
*B. contaminans* LMG 23,361	50.5	[47.1—53.9]	44.6	[42.1—47.2]	49.3	[46.3—52.4]	0.47
*B. metallica* LMG 24,068	54.7	[51.1—58.1]	44.5	[42.0—47.1]	52.9	[49.8—55.9]	0.7
*B. diffusa* CCUG 54,558	53.4	[49.9—56.8]	41.1	[38.6—43.6]	50.8	[47.7—53.8]	0.09
*B. ambifaria* AMMD	52.5	[49.0—55.9]	40.1	[37.6—42.6]	49.7	[46.7—52.8]	0.42
*B. stagnalis* CCUG 65,686	41.6	[38.2—45.0]	34.4	[32.0—36.9]	39.3	[36.3—42.3]	0.68

We also used the TrueBac™ ID [31] to make genome-wide alignment, and found *Burkholderia* strain S-53 has the highest similarity to *Burkholderia pyrrocinia*, and *Burkholderia stabilis* (Table 5). Its taxonomic ranks include Bacteria, Proteobacteria, Betaproteobacteria, Burkholderiales, Burkholderiaceae and *Burkholderia*.

**Table 5:** Whole genome alignment using TrueBac™ ID system

Hit Taxon	ANI (%)	ANI Coverage (%)	16S (%)	recA (%)	rplc (%)
*B. pyrrocinia*	95.99	82.7	99.86	98.88	99.39
*B. stabilis*	93.13	72.4	99.86	96.55	98.46
*QWEX_s*	93.75	75.7	99.86	97.39	99.08
*CP013402_s*	92.27	73.7	99.66	N/A	98.16
*B. contaminans*	92.20	63.9	99.59	N/A	98.16
*B. stagnalis*	89.35	60.6	99.59	96.27	N/A
*JJOA_s*	92.41	72.7	99.59	96.28	98.31
*B. ambifaria*	91.10	70.8	99.59	N/A	N/A
*CP003774_s*	91.60	71.8	99.52	96.55	N/A
*B. cepacia*	92.48	74.3	99.52	96.92	N/A
*B. paludis*	92.08	66.1	99.45	N/A	98.46
*B. cenocepacia*	92.41	71.6	99.45	N/A	98.46
*CP024904_s*	93.43	77.7	99.24	N/A	98.77
*B. vietnamiensis*	90.03	60.2	99.18	N/A	N/A
*PTXL_s*	89.27	61.0	99.18	N/A	N/A

### Phylogenetic analysis via GBDP method

Using Genome BLAST Distance Phylogeny (GBDP) method and tree builder service, the phylogeny tree of *Burkholderia* strain S-53 using its whole genome sequence was created while FastME was used to estimate the tree using GBDP intergenomic distances derived from complete proteomes.

**Fig. 3:**
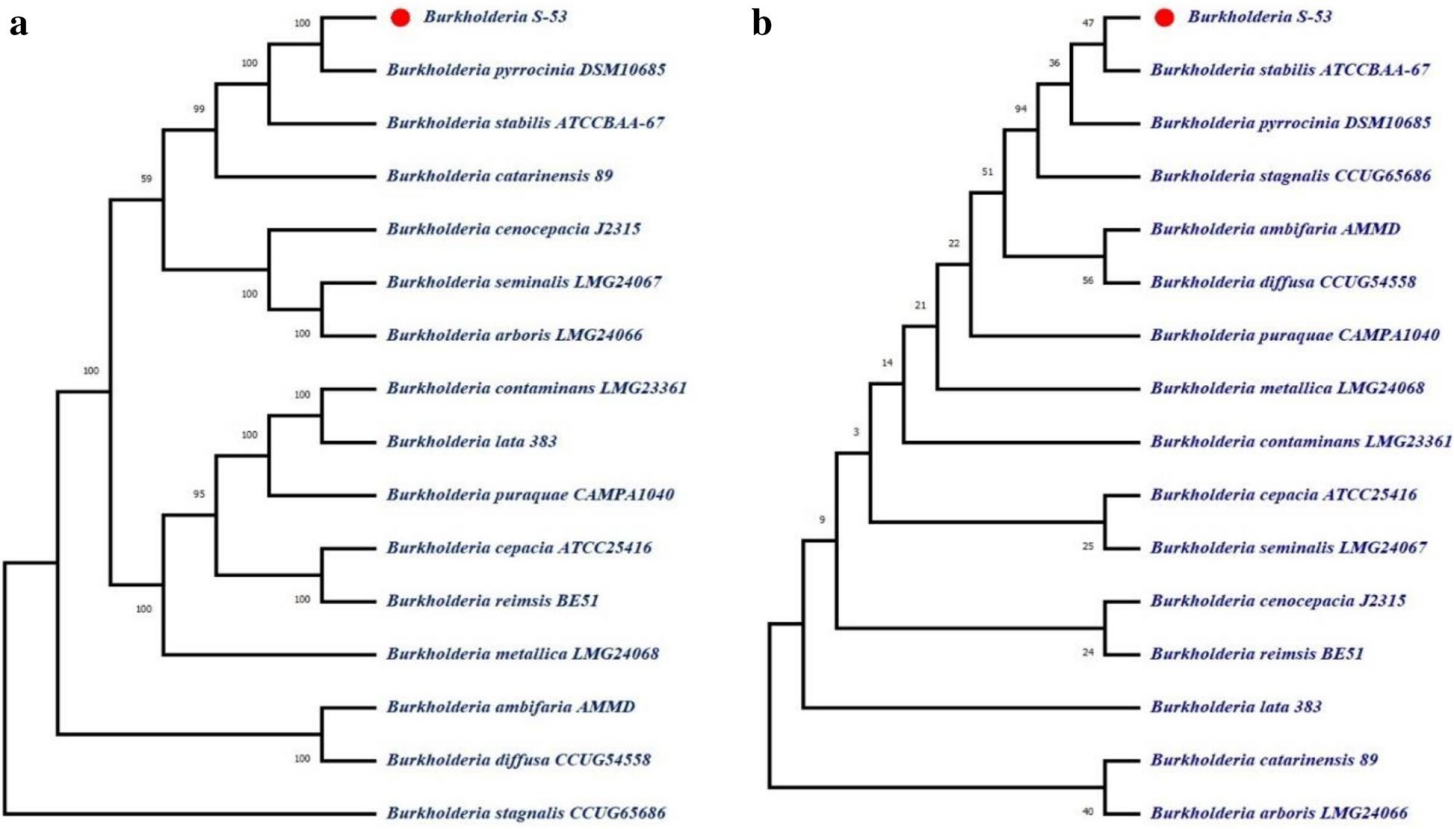
GBDP phylogenetic trees of S-53. The Genome BLAST Distance Phylogeny (GBDP) method was used to estimate the phylogeny. GBDP intergenomic distances computed from complete proteomes were used to estimate the tree using FastME. Using 16S rRNA sequences (**a**) or whole genome sequences (**b**), GBDP phylogenetic trees were constructed

GBDP phylogenetic tree constructed by using 16S rRNA indicated that S-53 is similar to *Burkholderia pyrrocinia* DSM10685K (Fig. 3a). On the other hand, GBDP phylogenetic tree constructed by using whole genome indicated that S-53 is similar to *B. stabilis* ATCCBAA-67 (Fig. 3b).

In all, 16S rRNA-based GBDP phylogenetic tree and whole genome alignment and comparative genome analysis suggested it to be *Burkholderia pyrrocinia*, while GBDP phylogenetic tree constructed by using whole genome supported it to be *B. stabilis*. Combining these analyses, we concluded it to be closer to *Burkholderia pyrrocinia.*

**Fig. 4:**
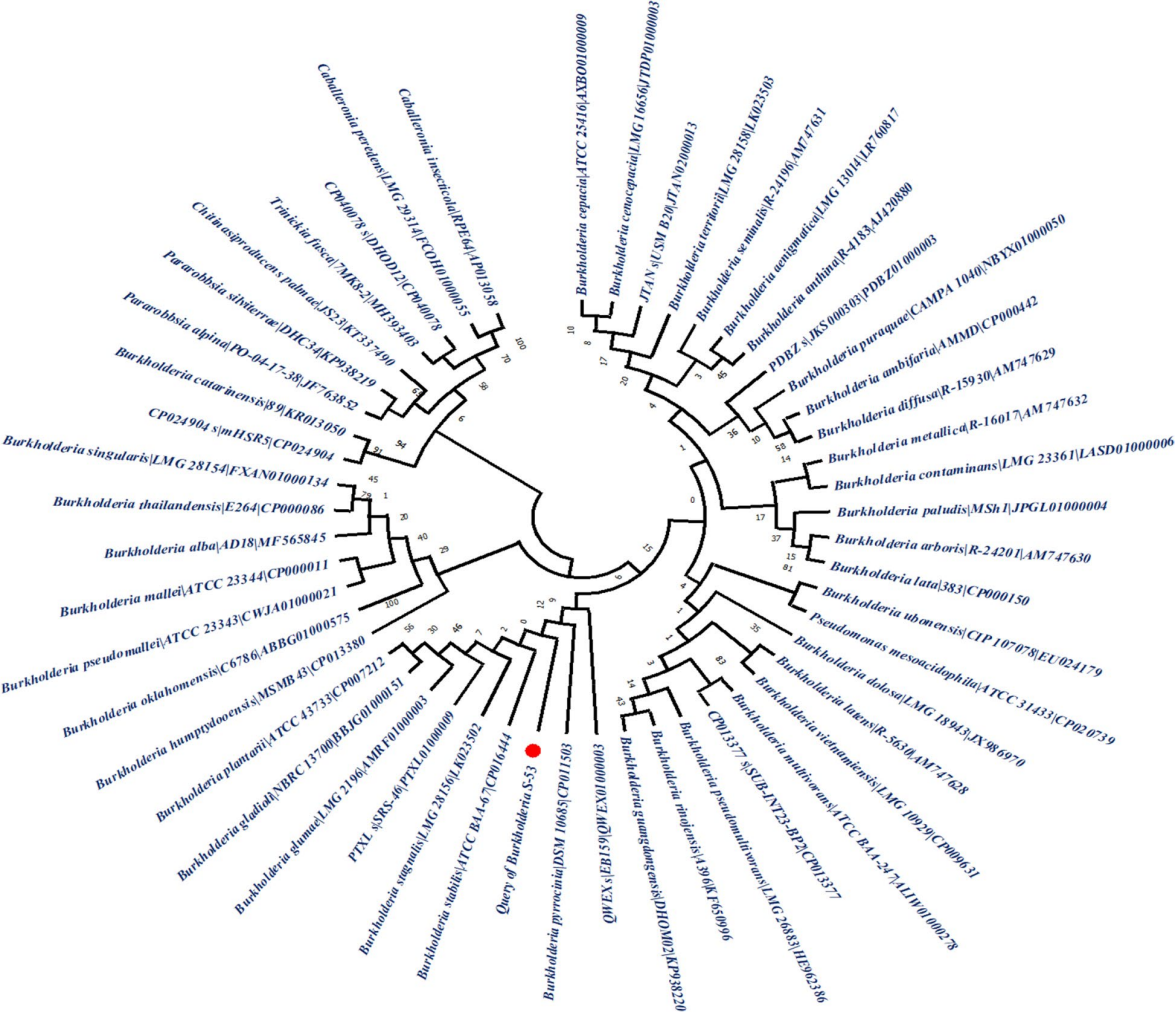
Evolutionary analysis of S-53 using the Maximum Likelihood method. The proportion of phylogenetic trees with the same taxonomy is given next to the branches. For the heuristic search, we used Neighbor-Join and BioNJ algorithms on a matrice of pairwise distances evaluated using Maximum Composite Likelihood (MCL) and chose the topology with the best log likelihood value. The tree's branch lengths are measured in substitutions per location. These 51 nucleotides were studied. Gaps and incomplete data were removed from all spots (complete deletion option). The final dataset has 1279 positions. MEGA X was used to study evolution

On the other hand, the phylogenetic tree was constructed from EzBioCloud 16S database by maximum-likelihood methods by Mega X application with 100 bootstrap values depicted in the Fig. 4. According to the maximum likelihood method, S-53 is close to the *Burkholderia stabilis* ATCC BAA-67 and *Burkholderia pyrrocinia* DSM 10,685.

### Prediction of NP BGCs in S-53 genome

Using antiSMASH 6.0 [36], BAGEL 4 [48] and PRISM 4 [49], we found a lot of BGCs on the *Burkholderia* strain S-53 genome for different secondary metabolites.

**Fig. 5:**
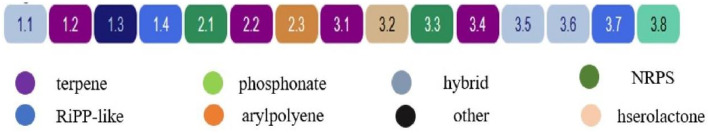
Localization of secondary metabolite clusters in the genome of *Burkholderia* sp. S-53

Through the prediction using antiSMASH 6.0, 15 BGCs were discovered (Table 6 and Fig. 5). The major BGC types include those for NRPs (2 BGCs), terpene (4GCs) and hybrid (3 BGCs) (Fig. 5).

**Table 6:** The analysis of biosynthetic pathways in *Burkholderia* sp. S-53 by antiSMASH 6.0

Cluster serial number	Region	Type^*^	From	To	Most similar known cluster	^#^Similarity
1	1.1	transAT-PKS,butyrolactone	142,912	228,926	lactimidomycin/8,9-dihydrolactimidomycin/8-hydr oxy-8,9-dihydrolactidomycin/7-hydroxy-8-desmetho y-isomigrastatin	44%
2	1.2	terpene	355,571	377,625		
3	1.3	other	742,229	783,314	pyrrolnitrin	100%
4	1.4	RiPP-like	963,741	974,556		
5	2.1	NRPS	1,804,138	1,858,813	ornibactin	100%
6	2.2	terpene	2,235,266	2,256,099		
7	2.3	arylpolyene	3,200,509	3,241,720	APE Vf	10%
8	3.1	terpene	44,410	63,612	N-acyloxyacyl glutamine	50%
9	3.2	hserlactone	622,283	642,891		
10	3.3	NRPS	979,395	1,029,254	pyochelin	100%
11	3.4	terpene	1,728,924	1,753,023		
12	3.5	siderophore,RRE-containing,RiPP-like	1,777,030	1,809,926	staphylobactin	18%
13	3.6	NRPS-like,betalactone	1,976,594	2,020,151	fragin	87%
14	3.7	RiPP-like	2,375,696	2,386,586		
15	3.8	phosphonate	3,223,192	3,264,880		

^*^NRPS**-** Non ribosomal peptide synthetase cluster

^#^The “similarity” is the percent of homologous genes in the query and hit clusters. As defined by antiSMASH, the homologous genes were chosen for their high sequence identity (> 30%) and short BLAST alignments (> 25%)

Among 15 BGCs, five BGCs (cluster 3, 5, 8, 10 and 13 regions 1.3, 2.1, 3.1, 3.3 and 3.6) were more than 50% identical to known BGCs. Other BGCs exhibited just a low degree of similarity or resemblance to previously identified BGCs, implying that *Burkholderia* sp. S-53 has a significant potential for the production of novel NPs in the future.

Moreover, we performed BAGEL analysis on S-53 genome and identified additional 2 different clusters for bacteriocins and RiPPs (Table 7).

**Table 7:** Clusters in S-53 for RiPP and bacteriocin predicted by BAGEL

AOI	Start	End	Class
S53_1_chr2_pilon.0.AOI_01	912,170	932,818	87.3; putidacin_L1
S53_1_chr1_pilon.1.AOI_01	958,742	979,501	81.3; Linocin_M18Bacteriocin

In addition, PRISM algorithm (https://prism.adapsyn.com/results/4c5c8259bfef7b827d3c7b9cdc95df6c) was used here to predict the structures of genetically encoded natural products using *Burkholderia* sp. S-53 genomes.

**Fig. 6:**
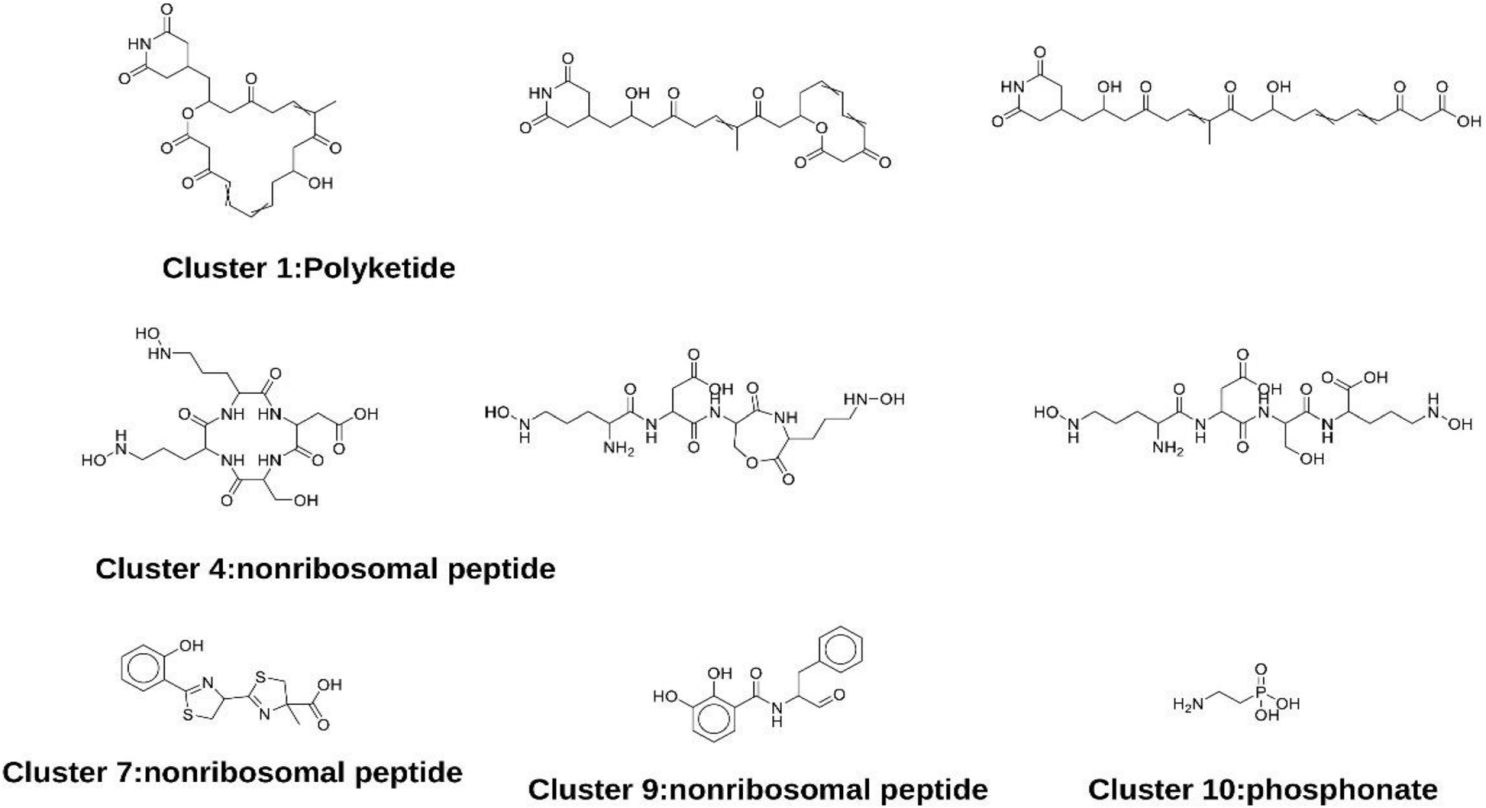
Predicted structures of secondary metabolites by S-53 using PRISM

Figure 6 showed predicted compounds by a total 10 clusters, including 3 for NRPs, 2 for PKs, 1 for Class II/III bacteriocin, 1 for aryl polyene and 1 for acyl homoserine lactone (Fig. 6).

## Discussion and conclusion

The high potential of *Burkholderia* to produce bioactive NPs has been reported with an increasing publishing record in decade years. Moreover, the rapid growth rate and low fermentation cost make them as a potential host for heterologous expression of some NP BGCs, aided by the establishment of genetic manipulation systems [50].

In this work, genomic investigation of microbes isolated from underexplored mountain habitats found several strains of *Burkholderia* species, among of which, S-53 attracted us for its comparatively quicker growth rate (16–18 h for entering stationary stage, compared to 24 h for general *Burkholderia* species), as a critical feature when it could be developed into a host for expressing some NP BGCs later.

We identified it using different methods and analyzed its evolution. Given that modern bacterial taxonomy uses genome sequence data to identify taxa, by means of genome sequence data, identification of a bacterial species is considered always to be more correct and persuasive. Thus, though different molecular methods for bacterial identification gave a little different result, our data deduced it to be *Burkholderia pyrrocinia*.

Other subspecies of *Burkholderia pyrrocinia* were ever isolated from different habitats, such as *Burkholderia pyrrocinia* JK-SH007, a plant growth-promoting bacteria from plat rhizosphere [51]. *Burkholderia pyrrocinia,* along with *Burkholderia cenocepacia* and *Burkholderia ambifari,* was referenced as *Burkholderia cepacia* complex (BCC) species, which are most frequently associated with roots of crop plants [52].

Taxonomy and identification of species in *Burkholderia* still are quite challenging. Though a high similarity of 16S rDNA ranging 98–100% often is used as “common standard” for bacterial identification at species level, it could not be applicable to classification of *Burkholderia* species [53], especially, for classification of BCC group of *Burkholderia*. So, the whole-genome-sequence-based taxonomic analysis could give comparably more reliable results, when combining other molecular methods.

Genomics-based bottom-up techniques have been developed to reveal previously undiscovered natural product biosynthesis pathways [54]. Here, whole genome sequencing and bioinformatic analyses of *Burkholderia* strain S-53 revealed many secondary metabolite biosynthetic gene clusters. Moreover, bioinformatics analysis uncovered more than two-thirds of BGCs in S-53 are not related to recognized clusters (Table 6).

These data supported that S-53 could be a good candidate used for identifying new NPs. Next, more research is needed to improve, isolate, and identify new bioactive natural products from this strain and to investigate the possibility of it to be as chassis for expressing of new NPs.

## Data Availability

The partial 16S rDNA gene sequence and genome sequence of the S-53 strain was deposited in the GenBank nucleotide database with an accession number of OM019084 and CP090482-CP090484.

## References

[1] A. Resistance, “Tackling a Crisis for the Health and Wealth of Nations,” Rev. Antimicrob. Resist., 2014.

[2] Toner E, Adalja A, Gronvall GK, Cicero A, Inglesby TV (2015). Antimicrobial resistance is a global health emergency. Heal Secur.

[3] Genilloud O (2014). The re-emerging role of microbial natural products in antibiotic discovery. Antonie Van Leeuwenhoek.

[4] Hutchings MI, Truman AW, Wilkinson B (2019). Antibiotics: past, present and future. Curr Opin Microbiol.

[5] Katz L, Baltz RH (2016). Natural product discovery: past, present, and future. J Ind Microbiol Biotechnol.

[6] R. D. Firn and C. G. Jones, “An explanation of secondary product ‘redundancy,’” in Phytochemical diversity and redundancy in ecological interactions, Springer, 1996, pp. 295–312.

[7] Galanie S, Entwistle D, Lalonde J (2020). Engineering biosynthetic enzymes for industrial natural product synthesis. Nat Prod Rep.

[8] K. Alam, J. Hao, Y. Zhang, and A. Li, “Synthetic biology-inspired strategies and tools for engineering of microbial natural product biosynthetic pathways,” Biotechnol. Adv., p. 107759, 2021. 10.1016/j.biotechadv.2021.107759 33930523

[9] C. L. Schoch et al., “NCBI Taxonomy: a comprehensive update on curation, resources and tools,” Database, vol. 2020, 2020. 10.1093/database/baaa062 PMC7408187 32761142

[10] Depoorter E, Bull MJ, Peeters C, Coenye T, Vandamme P, Mahenthiralingam E (2016). Burkholderia: an update on taxonomy and biotechnological potential as antibiotic producers. Appl Microbiol Biotechnol.

[11] Kunakom S, Eustáquio AS (2019). Burkholderia as a source of natural products. J Nat Prod.

[12] Alam K (2022). In silico genome mining of potential novel biosynthetic gene clusters for drug discovery from Burkholderia bacteria. Comput Biol Med.

[13] Liu X, Cheng Y-Q (2014). Genome-guided discovery of diverse natural products from Burkholderia sp. J Ind Microbiol Biotechnol.

[14] Hwang S (2019). Primary transcriptome and translatome analysis determines transcriptional and translational regulatory elements encoded in the Streptomyces clavuligerus genome. Nucleic Acids Res.

[15] Li Y, Zhang C, Liu C, Ju J, Ma J (2018). Genome sequencing of Streptomyces atratus SCSIOZH16 and activation production of nocardamine via metabolic engineering. Front Microbiol.

[16] E. W. Myers et al., “A whole-genome assembly of Drosophila,” Science (80-. )., vol. 287, no. 5461, pp. 2196–2204, 2000. 10.1126/science.287.5461.2196 10731133

[17] J. C. Venter et al., “The sequence of the human genome,” Science (80-. )., vol. 291, no. 5507, pp. 1304–1351, 2001. 10.1126/science.1058040 11181995

[18] Istrail S (2004). Whole-genome shotgun assembly and comparison of human genome assemblies. Proc Natl Acad Sci.

[19] Levy S (2007). The diploid genome sequence of an individual human. PLoS Biol.

[20] Goldberg SMD (2006). A Sanger/pyrosequencing hybrid approach for the generation of high-quality draft assemblies of marine microbial genomes. Proc Natl Acad Sci.

[21] Berlin K, Koren S, Chin C-S, Drake JP, Landolin JM, Phillippy AM (2015). Assembling large genomes with single-molecule sequencing and locality-sensitive hashing. Nat Biotechnol.

[22] Delcher AL, Bratke KA, Powers EC, Salzberg SL (2007). Identifying bacterial genes and endosymbiont DNA with Glimmer. Bioinformatics.

[23] Stanke M, Schöffmann O, Morgenstern B, Waack S (2006). Gene prediction in eukaryotes with a generalized hidden Markov model that uses hints from external sources. BMC Bioinformatics.

[24] Lowe TM, Eddy SR (1997). tRNAscan-SE: a program for improved detection of transfer RNA genes in genomic sequence. Nucleic Acids Res.

[25] G. O. Consortium, “The Gene Ontology (GO) database and informatics resource,” Nucleic Acids Res., vol. 32, no. suppl_1, pp. D258–D261, 2004. 10.1093/nar/gkh036 PMC308770 14681407

[26] Kanehisa M, Goto S (2000). KEGG: kyoto encyclopedia of genes and genomes. Nucleic Acids Res.

[27] Meier-Kolthoff JP, Göker M (2019). TYGS is an automated high-throughput platform for state-of-the-art genome-based taxonomy. Nat Commun.

[28] Yoon S-H (2017). Introducing EzBioCloud: a taxonomically united database of 16S rRNA gene sequences and whole-genome assemblies. Int J Syst Evol Microbiol.

[29] Felsenstein J (1981). Evolutionary trees from DNA sequences: a maximum likelihood approach. J Mol Evol.

[30] Kumar S, Stecher G, Li M, Knyaz C, Tamura K (2018). MEGA X: molecular evolutionary genetics analysis across computing platforms. Mol Biol Evol.

[31] Ha S-M (2019). Application of the whole genome-based bacterial identification system, TrueBac ID, using clinical isolates that were not identified with three matrix-assisted laser desorption/ionization time-of-flight mass spectrometry (MALDI-TOF MS) systems. Ann Lab Med.

[32] Camacho C (2009). BLAST+: architecture and applications. BMC Bioinformatics.

[33] Lee I, Kim YO, Park S-C, Chun J (2016). OrthoANI: an improved algorithm and software for calculating average nucleotide identity. Int J Syst Evol Microbiol.

[34] L. M. Rodriguez-R and K. T. Konstantinidis, “The enveomics collection: a toolbox for specialized analyses of microbial genomes and metagenomes,” PeerJ Preprints, 2016.

[35] Yoon S-H, Ha S-M, Lim J, Kwon S, Chun J (2017). A large-scale evaluation of algorithms to calculate average nucleotide identity. Antonie Van Leeuwenhoek.

[36] K. Blin et al., “antiSMASH 6.0: improving cluster detection and comparison capabilities,” Nucleic Acids Res., p. 1, 2021. 10.1093/nar/gkab335 PMC8262755 33978755

[37] Machado H, Sonnenschein EC, Melchiorsen J, Gram L (2015). Genome mining reveals unlocked bioactive potential of marine Gram-negative bacteria. BMC Genomics.

[38] Churchill GA (1989). Stochastic models for heterogeneous DNA sequences. Bull Math Biol.

[39] Altschul SF, Gish W, Miller W, Myers EW, Lipman DJ (1990). Basic local alignment search tool. J Mol Biol.

[40] Finn RD (2014). Pfam: the protein families database. Nucleic Acids Res.

[41] D. A. Benson et al., “GenBank Nucleic Acids Res 41 (D1),” D36–D42, 2013. 10.1093/nar/gkw1070 PMC5210553 27899564

[42] U. Consortium (2015). UniProt: a hub for protein information. Nucleic Acids Res.

[43] R. Hammami, A. Zouhir, C. Le Lay, J. Ben Hamida, and I. Fliss, “BACTIBASE second release: a database and tool platform for bacteriocin characterization,” Bmc Microbiol., vol. 10, no. 1, pp. 1–5, 2010. 10.1186/1471-2180-10-22 PMC2824694 20105292

[44] Waghu FH, Barai RS, Gurung P, Idicula-Thomas S (2016). CAMPR3: a database on sequences, structures and signatures of antimicrobial peptides. Nucleic Acids Res.

[45] Medema MH (2015). Minimum information about a biosynthetic gene cluster. Nat Chem Biol.

[46] Ziemert N, Podell S, Penn K, Badger JH, Allen E, Jensen PR (2012). The natural product domain seeker NaPDoS: a phylogeny based bioinformatic tool to classify secondary metabolite gene diversity. PLoS ONE.

[47] J. R. Grant and P. Stothard, “The CGView Server: a comparative genomics tool for circular genomes,” Nucleic Acids Res., vol. 36, no. suppl_2, pp. W181–W184, 2008. 10.1093/nar/gkn179 PMC2447734 18411202

[48] van Heel AJ, de Jong A, Song C, Viel JH, Kok J, Kuipers OP (2018). BAGEL4: a user-friendly web server to thoroughly mine RiPPs and bacteriocins. Nucleic Acids Res.

[49] Skinnider MA (2020). Comprehensive prediction of secondary metabolite structure and biological activity from microbial genome sequences. Nat Commun.

[50] Liu J (2021). Rational construction of genome-reduced Burkholderiales chassis facilitates efficient heterologous production of natural products from proteobacteria. Nat Commun.

[51] W.-H. Liu et al., “Indole-3-acetic acid in Burkholderia pyrrocinia JK-SH007: Enzymatic identification of the indole-3-acetamide synthesis pathway,” Front. Microbiol., p. 2559, 2019. 10.3389/fmicb.2019.02559 PMC6848275 31749788

[52] Alisi C (2005). Metabolic profiling of Burkholderia cenocepacia, Burkholderia ambifaria, and Burkholderia pyrrocinia isolates from maize rhizosphere. Microb Ecol.

[53] Sfeir MM (2018). Burkholderia cepacia complex infections: more complex than the bacterium name suggest. J Infect.

[54] Winter JM, Behnken S, Hertweck C (2011). Genomics-inspired discovery of natural products. Curr Opin Chem Biol.

